# Diversity in Viral Anti-PKR Mechanisms: A Remarkable Case of Evolutionary Convergence

**DOI:** 10.1371/journal.pone.0016711

**Published:** 2011-02-02

**Authors:** Elena Domingo-Gil, René Toribio, José Luis Nájera, Mariano Esteban, Iván Ventoso

**Affiliations:** 1 Departamento de Biología Molecular and Centro de Biología Molecular “Severo Ochoa” (CSIC-UAM), Universidad Autónoma de Madrid, Madrid, Spain; 2 Centro Nacional de Biotecnología (CSIC), Campus Universidad Autónoma de Madrid, Madrid, Spain; Institut Pasteur, France

## Abstract

Most viruses express during infection products that prevent or neutralize the effect of the host dsRNA activated protein kinase (PKR). Translation of Sindbis virus (SINV) mRNA escapes to PKR activation and eIF2 phosphorylation in infected cells by a mechanism that requires a stem loop structure in viral 26S mRNA termed DLP to initiate translation in the absence of functional eIF2. Unlike the rest of viruses tested, we found that Alphavirus infection allowed a strong PKR activation and eIF2α phosphorylation *in vitro* and in infected animals so that the presence of DLP structure in mRNA was critical for translation and replication of SINV. Interestingly, infection of MEFs with some viruses that express PKR inhibitors prevented eIF2α phosphorylation after superinfection with SINV, suggesting that viral anti-PKR mechanisms could be exchangeable. Thus, translation of SINV mutant lacking the DLP structure (ΔDLP) in 26S mRNA was partially rescued in cells expressing vaccinia virus (VV) E3 protein, a known inhibitor of PKR. This case of heterotypic complementation among evolutionary distant viruses confirmed experimentally a remarkable case of convergent evolution in viral anti-PKR mechanisms. Our data reinforce the critical role of PKR in regulating virus-host interaction and reveal the versatility of viruses to find different solutions to solve the same conflict.

## Introduction

During virus-host coevolution, the acquisition of an antiviral pathway by the host was generally followed by the appearance of a viral countermeasure so that the current host-parasite interactions are keeping on a dynamic equilibrium (the Red Queen principle) [1], [2], [3]. A paradigmatic example of these complex interactions are the different mechanisms that viruses have evolved to evade or subvert the antiviral effect of interferons (IFNs) and other pro-inflammatory cytokines that are secreted by vertebrate cells in response to virus and other pathogens [4], [5], [6], [7], [8], [9]. Thus, viruses express products that impair the detection of viral proteins or nucleic acids by host pattern-recognition receptors (PRRs; Toll-like receptors and RIG-I-like receptors), block the signaling pathways that lead to the synthesis of IFNα/β and other cytokines, or prevent the activation of some IFN-stimulated genes such as dsRNA-activated kinase (PKR) [6], [9], [10], [11], [12], [13], [14]. Moreover, in some cases such as poxvirus or HCV, one or more viral product can interfere with IFN secretion or signalling at multiple points, ensuring a more efficient viral escape to innate immunity of the host [6], [9].

**Figure 1:**
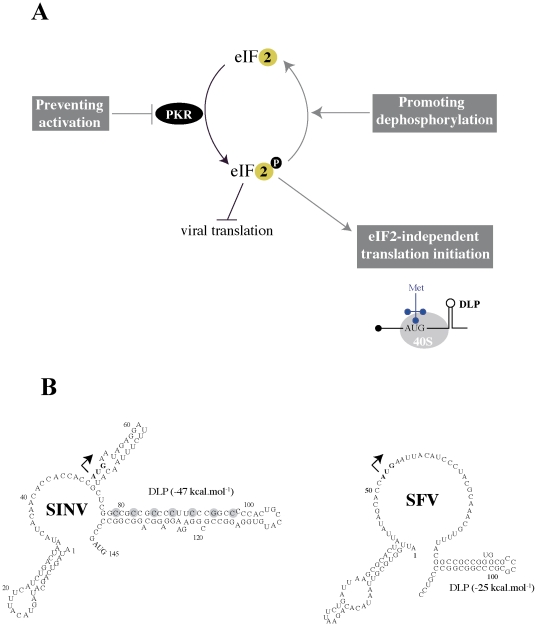
Current known viral strategies to prevent or counteract PKR activation can be grouped into three categories. *A*) First, viruses can prevent the activation of PKR by expressing proteins or RNAs that bind the kinase in an inhibitory manner, by sequestering dsRNA activator molecules or by inducing the degradation of PKR. Examples of this group are vaccinia E3 and K3, influenza NS1 and Adenovirus VAI RNAs and Rift Valley virus NS1 protein. In the second category are viruses that promote eIF2α dephosphorylation such as Herpes virus γ34.5 protein which acts as a regulatory subunit of cellular protein phosphatase 1a (PP1). Herpesvirus also express products from the first group (Us11 gene, a PKR inhibitor). In the third category, some viruses do not prevent PKR-mediated eIF2α phosphorylation, but viral mRNA can initiate translation in an eIF2-independent manner by means of a RNA structure (DLP) that stalls the scanning 40S ribosome on the initiation codon. To date, this strategy has been found only in Alphavirus. *B*) Secondary structure prediction of the 5′extreme of 26S mRNAs of SINV and SFV. The stability of DLP structure (ΔG°) is expressed in kcal. mol^−1^. Arrows show the initiation codon. Circled nucleotides in SINV were mutated to adenines in ΔDLP mutant virus as described previously [29].

Among innate antiviral mechanisms of vertebrates, PKR activation constitutes one of the first line of antiviral defense acting at the immediate-early phase of virus replication that precedes the eventual secretion of IFN. PKR is present at basal levels in most of mammalian tissues, but its amount increases after priming cells with IFNα/β [15], [16]. PKR binds dsRNA molecules generated during the replication of RNA viruses as well as in some transcripts from DNA viruses, leading to the activation of the kinase by a sequential wave of autophosphorylation events [17], [18], [19], [20]. Activated PKR phosphorylates and inactivates eukaryotic initiation factor 2 (eIF2), the only well-described substrate of the kinase that is also phosphorylated by other members of eIF2-kinase family in vertebrates [17], [21]. As result of this, the general translation is rapidly inhibited in an attempt of the infected cells to block viral translation and abort virus spreading [16], [22]. However, as in other pathways of innate response, viruses have evolved a variety of strategies to prevent or overcome the activation of PKR in infected cells (reviewed in [12]). Among these mechanisms, the most frequent found are viral products that prevent the activation of PKR by sequestering its activator (dsRNA), by direct binding to the regulatory element of the kinase or by expressing a pseudosubstrate that competes with eIF2 for binding to the kinase (see figure 1). Other viruses such as poliovirus and Rift Valley virus induce the degradation of PKR by a mechanism that has not been well characterized yet [23], [24]. In some cases such as Herpes virus-infected cells, eIF2 phosphorylation is rapidly reversed by the action of viral phosphatases that are expressed along the infection [25], [26], [27]. For Alphavirus, the strategy is markedly different; PKR is strongly activated upon infection with SINV and Semliki Forest (SFV) viruses, so that eIF2 factor is completely phosphorylated [28], [29]. Translation of viral subgenomic mRNAs (26S), however, resists due to the presence of a prominent hairpin loop structure in mRNA located downstream of the initiation codon (DLP) that allows the 40S ribosome to initiate in the absence of eIF2 [29].

The existence of these different mechanism stress the critical role of PKR in regulating host-virus interaction and show a remarkable example of convergent evolution to generate different patterns of molecular mimicry [8]. For example, VV K3L mimics eIF2α acting as a competitive inhibitor of PKR, whereas Adenovirus VAI RNAs mimics dsRNA activators of PKR by binding the kinase in an inhibitory manner [8], [12]. Fingerprints of this antagonist coevolution has been found recently in the accelerated rates of positive evolution found in PKR gene for defeating viral anti-PKR products such as poxvirus E3 and K3 [30], [31]. However, an important issue that has not been addressed to date is whether these different anti-PKR mechanisms might be exchanged between non related viruses to get a functional complementation. In this work we show that a cis-acting RNA structure of Alphaviruses responsible for counteracting PKR activation can be replaced by a viral protein such as vaccinia E3.

## Results

### Alphavirus are unique in allowing PKR activation in cultured cells and infected animals

**Figure 2:**
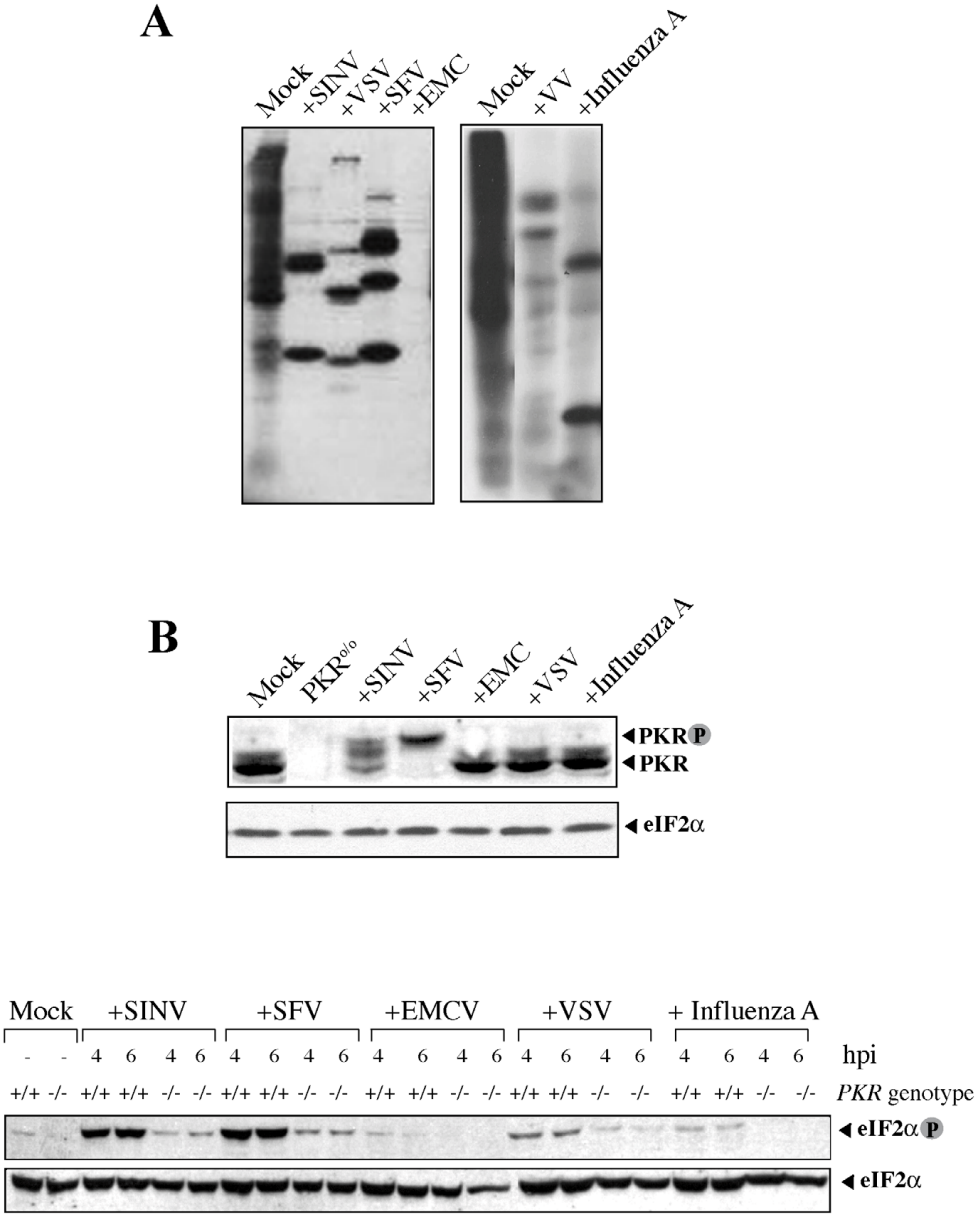
Alphaviruses are unique in allowing PKR activation and eIF2α phosphorylation in infected cells. *A*) Susceptibility of MEFs to viruses used in this study. Cells were infected with the indicated viruses to a moi of 25 pfu/cell and 6 h later pulsed with [^35^S]-Met for 30 min. Labeled proteins were resolved by SDS-PAGE followed of autoradiography. *B*) PKR activation and eIF2α phosphorylation in wild type or *PKR* ^o/o^ MEFs infected with the indicated viruses at 4 and 6 hpi. Note the mobility shift of PKR band upon activation in SINV and SFV-infected cells (upper panel). eIF2α phosphorylation in wild type (+/+) and *PKR* knock-out cells (o/o) infected with the indicated viruses. Only Alphavirus-infected cultures showed a strong eIF2α phosphorylation. For VSV-infected cells, a slight increase in eIF2α phosphorylation was also observed.

We and others have reported before that infection of 3T3 fibroblasts with SINV virus triggered the complete phosphorylation of eIF2 due to a strong PKR activation [28], [29] (Figure 2). We wanted to test whether such phosphorylation of host eIF2α was also detected in cells infected with other Alphaviruses and with members of other unrelated families of RNA viruses. For this, we infected cells with SFV (Alphavirus), VSV (Rhabdovirus), Influenza A (Orthomyxovirus) and EMCV (Picornavirus) (Figure 2A). Equivalent or even stronger PKR activation was observed in cells infected with SFV as judged by the mobility shift of PKR band in SDS-PAGE that was indicative of autophosphorylation associated to kinase activation (Figure 2B). As for SINV, activation of PKR in SFV-infected cells induced a strong phosphorylation of eIF2α that was not observed in the rest of viruses analyzed with the exception of VSV, where a slight increase in eIF2 phosphorylation was observed as reported before [16]. The use of *PKR* gene knock-out cells (*PKR* ^o/o^) showed that eIF2 phosphorylation in Alphavirus-infected cells was almost completely attributable to PKR activation as described before (Figure 2B and [29]. With the exception of Alphavirus, the rest of RNA viruses tested here did not trigger a substantial activation of eIF2 kinases in infected cells.

**Figure 3:**
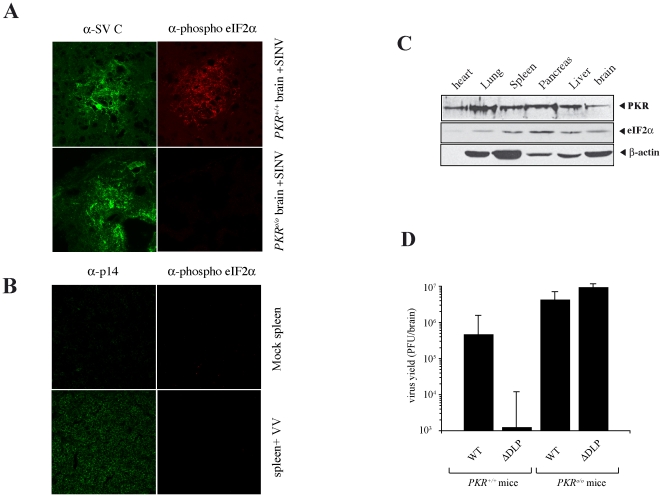
eIF2α phosphorylation and translational resistance of SINV virus also operates in infected animals. *A*) IF analysis of brains from wild type and *PKR* knock-out mice infected with SINV at 4 dpi. Adjacent sections were incubated with anti-SINV and anti-phosphoeIF2α antibodies as described in Materials and Methods. *B*) IF analysis of spleens from mice infected with VV-Luc at 1dpi. Sections were incubated with anti-VVp14 (reactive against the envelope protein A27) and anti-phosphoeIF2α antibodies. Note that spleen cells expressing viral antigens did not react with anti- phosphoeIF2α antibodies. *C*) Expression of *PKR* in different mouse organs from uninfected animals. Equivalent amounts of protein extracts were analyzed by immunoblot against PKR, total eIF2α and β-actin. *D*) Attenuation of ΔDLP mutant virus in wild type, but not in *PKR* ^o/o^ mice. Animals were inoculated with 10^7^ of WT and 2×10^7^ of ΔDLP mutant viruses. Viral yields in mouse brains at 4 dpi were titrated by plaque assay. Results are the mean from 10 animals inoculated for each group in three independent experiments. SD from each group is also showed.

Next, we analyzed whether eIF2α phosphorylation was also detected in animals infected with Alphavirus. For this, mice were infected with SINV by the intranasal route, and 4 days later brains were subjected to immunofluorescence (IF) analysis. As described previously, SINV showed a marked tropism for cortical and spinal cord neurons [32], [33], [34], [35]. Groups of neurons expressing viral antigens were easily detected in pyriform, motor and somatosensorial areas of brain cortex as well in hippocampus. Notably, the vast majority of these infected cells showed a strong staining of phospho eIF2 (Figure 3A), with no signal detected outside of areas of viral replication. Clearly, no eIF2 phosphorylation was detected in infected neurons of *PKR* ^o/o^ mice, showing that as occurred *in vitro* PKR was responsible for eIF2 phosphorylation in response to infection of animals (Figure 3A). We extended this *in vivo* analysis to other viruses endowed with anti-PKR mechanisms such as VV. Clearly, no eIF2 phosphorylation was detected in spleens of mice infected with VV despite the efficient replication detected in this organ (Figure 3B). The absence of eIF2 phosphorylation in mice infected with VV was not attributable to a low expression of the kinase in spleen, since a comparable expression of PKR was detected in all murine organs analyzed (Figure 3C). Taken together, our results show that PKR-induced eIF2 phosphorylation seems to be a specific feature of animals infected with Alphavirus.

### Resistance to PKR activation is critical for replication of Alphavirus in animals

Viral anti-PKR products commonly act as virulence factors necesary for efficient viral replication and pathogenesis in animals [25], [36], [37], [38]. To test whether translational resistance of SINV virus to PKR activation was important for replication *in vivo*, we compared the replication of wild type virus and a SINV mutant lacking the DLP structure (ΔDLP) in 26S mRNA. This structure in RNA has been reported before to be essential for translation and virus replication in 3T3 cells [29]. Clearly, replication of ΔDLP virus was greatly hampered in wild type, but not in *PKR* ^o/o^ mice (Figure 3D). About 3–4 log reduction in viral yield of ΔDLP virus were found in *PKR* ^+/+^ mice (wild type) when compared to *PKR* ^o/o^ counterparts, whereas wild type viruses replicated at similar levels in both animal types. This result shows that as predicted from other viruses, PKR resistance in Alphavirus is essential for efficient replication *in vivo*.

### Rescue of SINV ΔDLP translation by poxvirus E3 protein

**Figure 4:**
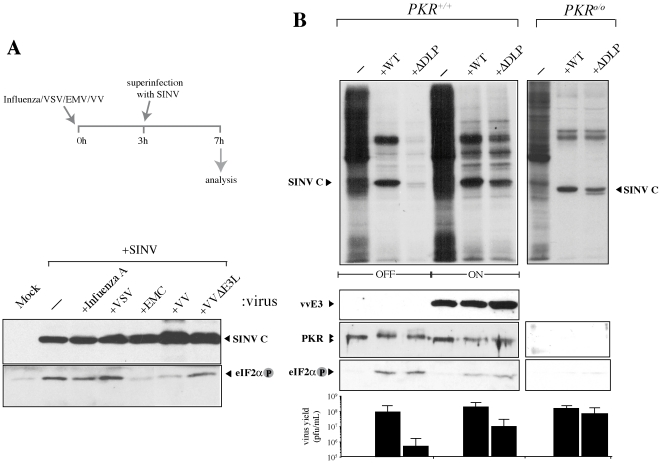
Anti-PKR mechanisms can be exchanged between viruses. *A*) Previous infection with some viruses prevented eIF2α phosphorylation after SINV virus superinfection. The protocol followed for mixed infections is outlined. Wild type MEFs were infected with the indicated viruses at a moi of 2 pfu/cell. Three hours later, cells were superinfected with SINV at moi of 25 pfu/cell and 5 h later lysed in sample buffer for immunoblot analysis against SINV capsid (SINV C, upper panel) and anti-phosphoeIF2α (bottom panel). *B*) Expression of VV E3L gene rescued translation of ΔDLP SINV mutant. MEF-E3L cells were induced for the expression of VV E3L by tetracycline withdrawal and infected with SINV-WT or SINV-ΔDLP mutant. Five hours later, cells were labeled with [^35^S]-Met+Cys for 30 min and analyzed by SDS-PAGE followed of autoradiography and by immunoblot against anti-E3, anti-PKR and anti-phospho eIF2α. Parallel cultures were infected at moi of 5 pfu/cell and viral yields were determined by plaque assay 2 days later. Data are the mean ±SD from three and two independent experiments in PKR^+/+^ and PKR^o/o^ cells, respectively.

In a first attempt to test whether anti-PKR mechanisms could be exchangeable among viruses, we analyzed the phosphorylation state of eIF2 in SINV-infected cells that had been first infected with low doses of some RNA (Influenza A, VSV and EMVC) and DNA (VV) viruses (Figure 4A). Time-lag between infections was set to allow the simultaneous expression of products from both viruses. Thus, the previous infection with the indicated viruses did not prevent the replication and accumulation of SINV proteins in doubly infected cells (Figure 4A). Interestingly, pre-infection with VV and EMCV viruses completely prevented eIF2 phosphorylation triggered by SINV superinfection. However, other viruses such as VSV were unable to prevent PKR activation after superinfection with SINV, whereas Influenza A infection exerted only a moderate preventive effect (∼50% reduction). In this experiment we also included a VV mutant lacking the E3L gene, the main PKR inhibitor in VV-infected cells [39], [40], [41]. Notably, mutant VV-ΔE3L was unable to prevent eIF2α phosphorylation after SINV superinfection, showing that the inhibitory potential of VV on PKR is largely attributable to the action of E3 protein. This trans-inhibitory activity of E3 opened the possibility to rescue translation of SINV ΔDLP mutant by expressing VV E3L gene in MEF cells. For this, we used a previous established MEF line that expressed the VV E3 protein in an inducible manner after tetracycline withdrawal [42]. As is shown is Figure 4B, induction of E3L gene partially prevented eIF2 phosphorylation after SINV infection. Moreover, mobility shift of PKR in SDS-PAGE was prevented by expression of E3 protein, showing that this viral product targeted PKR to block its activation. Notably, expression of E3 protein partially restored translation of ΔDLP virus mRNA that was strongly inhibited due to eIF2 phosphorylation in non induced cells. This allowed a ∼50 fold increase of ΔDLP virus yield in induced cells, showing that a deficiency in a cis-acting RNA structure involved in translation can be complemented by an unrelated product but that participates in the same biological trait.

## Discussion

Adaptation of non-related parasites to the same host or ecological niche is generally associated to events of convergent evolution (homoplasy), where analogous genetic elements arise during the acquisition of the same biological trait [8], [43]. A paradigmatic example of this is the plethora of viral mechanisms to counteract the innate response of vertebrates (reviewed in [6]). Collectively, viruses have acquired mechanisms to subvert or counteract virtually all the biochemical routes of the host that lead into the production of IFN and other antiviral cytokines (reviewed in [6], [8]). Among them, the appearance of mechanisms to prevent or counteract PKR activation likely increased the replication capabilities of viruses during their adaptation to vertebrate hosts. Thus, the type of anti-PKR mechanism found in a given virus seems to be greatly influenced by the genomic organization and the natural history of the virus during the adaptation to vertebrate hosts. Thus, the singular mechanism of PKR resistance found in Alphavirus probably reflects a peculiar evolutionary adaptation to vertebrates from arthropods (mainly mosquitoes), the vectors that Alphavirus and other Arbovirus use for transmission [35], [44], [45]. Moreover, the appearance of cis-acting RNA structures such as DLP to counteract PKR-mediated eIF2α phosphorylation was probably limited to simple monocistronic RNA virus such as Alphavirus, whereas in polycistronic RNA viruses (e.g. Influenza A) and in complex DNA viruses (e.g. Poxvirus) the acquisition of trans-acting PKR inhibitors allowed viral mRNAs to be collectively translated in infected cells. Although being functional equivalent, viral anti-PKR mechanisms that prevent or allow PKR activation could have different biological consequences. The strong eIF2α phosphorylation found in neurons of SINV-infected mice is enough to completely block translation of non-viral mRNAs throughout the infection [46], that probably leads to a rapid and irreversible death of infected cells. This early shut-off of host translation by eIF2α phosphorylation in SINV-infected cells could also suppress the synthesis of IFN and ISG as described recently for HCV infection [47]. This possibility agrees well with previous findings showing that infection with many Alphaviruses prevented the secretion of IFNα/β in infected cultured cells and animals, despite SINV showed an exquisite sensitivity to IFNα/β when cells were primed with this cytokine before infection [32], [48], [49]. For other viruses such as poxviruses, however, preventing PKR activation during *in vivo* replication could maintain protein synthesis in infected cells for more time, allowing the virus to express the late genes required for the culmination of the cycle [7], [36]. In this case, viruses had to further acquire a more specific mechanism to prevent the synthesis and secretion of interferons by blocking activation of NFkB or IRF7 factors [6], [9].

Our results show that, in addition to VV, EMCV expresses a trans-acting inhibitor that prevented the activation of PKR in response to SINV superinfection. However, unlike to that found in poliovirus-infected human cells, anti-PKR activity of EMCV does not seem to involve degradation of the kinase since no alteration in the steady state of PKR was found in EMCV-infected MEFs (Figure 2). For VSV, the absence of effect on SINV-triggered eIF2α phosphorylation was expected in part given the exquisite sensitivity that VSV showed to PKR activation and eIF2α phosphorylation. Thus, VSV seems to lack of an autonomous anti-PKR activity as suggested from previous results [16], suggesting that this virus could hide the replicative forms of dsRNA in a way that go unnoticed for host PKR [20]. For VV, our results show that E3 protein accounted for almost all the anti-PKR activity in doubly infected cells, stressing the main role of this protein over the K3 product that probably has only a marginal contribution to the anti-PKR activity of the virus as suggested before [39]. The heterotypic complementation obtained for SINV ΔDLP mutant in cells expressing E3 revealed a notable case of functional analogy that reinforced the importance of anti-PKR mechanisms for virus replication in vertebrates. Thus, as showed for other viruses such as VV, Herpes and Influenza A, elimination of anti-PKR mechanism drastically reduced the replication of SINV virus in wild type animals (Figure 4). However, SINV ΔDLP mutant replicated to wild type levels in *PKR* ^o/o^ mice where no eIF2α phosphorylation in infected neurons was detected. This result showed that cis-acting DLP structure in SINV 26S mRNA is only required to overcome the effect of eIF2α phosphorylation on translation of viral mRNA, in contrast with other scenarios such as Influenza NS1 or VV E3 proteins whose important functions extend beyond to PKR inhibition [36], [38], [42], [50]. At least for SINV virus, our results demonstrate that resistance to PKR is exerted exclusively at translational level and that probably only involves overcoming eIF2 phosphorylation. In our opinion this is a remarkable point because the existence of other substrates of PKR apart of eIF2α has been suggested before [51], [52] despite the exquisite catalytic specificity that PKR shows for eIF2 [17], [21].

Finally, the concept of molecular mimicry where pathogens have taken on proteins or protein domains from their hosts to gain evolutionary advantages could be also applicable to RNA structures such as DLP involved in translational control of some cellular mRNA. Thus, it is possible that Alphavirus had copied a mechanism of translation initiation that already operated in the cell. In our opinion, this is a very exciting possibility that deserves further investigation.

## Materials and Methods

### Ethics Statement

This study was carried out in strict accordance with the recommendations in the Directive 86/609/EEC of the European Union on the protection of animals used for experimental and other scientific purposes, and that was implemented by the Spanish Government under approval N° 1201/2005. The protocol was approved by the committee on the ethics of animal experiments of Universidad Autónoma de madrid (Permit Number: CEI 20-419). All surgery was performed under isoflurane anesthesia, and all efforts were made to minimize suffering.

### Cells, virus and infections

MEFs derived from wild type (C57BL6/129sv) and PKR^o/o^ knock-out mice was described previously [53]. MEFs expressing VV E3L gene under the control of tetracyclin repressor were described previously [42]. SINV, SFV, VSV (indiana strain), EMCV and VV-ΔE3L viruses were grown in BHK21. Influenza A virus (Victoria strain, a gift of Amelia Nieto, CNB, Madrid) and Vaccinia virus (VV and VV-Luc, [54]) were amplified in BSC-10 cells. SINV ΔDLP mutant bearing 7 point mutations that disrupted the secondary structure of downstream loop structure (DLP) in 26S mRNA was described previously [29]. Cells growing in 24 well plates were infected at the indicated multiplicity of infection (moi) in DMEN lacking serum for 1 h. After that, fresh medium containing 10% of calf or foetal serum was added. At the indicated times, cells were washed twice with cold DMEN lacking serum and lysed in sample buffer. To estimate viral yields, infections were done at a moi of 1–2 pfu/cell and two days later both medium and cells were recovered, frozen and thawed three times, centrifuged for 10 min at 8,000×g and virus in the supernatant was titrated in BHK21 cells.

### Infection of animals

4–6 week old mice of 129sv strain were infected with 10^7^ pfu of SINV by intranasal inoculation. *PKR* knock-out mice in the same genetic background were kindly provided by J.C. Bell (University of Ottawa, Canada) [55]. At 4–5 days postinfection, animals were sacrificed by cervical dislocation, decapitated and brain extracted. For VV inoculation, 5 weeks old mice of BALB/c strain were inoculated by the intraperitoneal route with 2×10^7^ pfu of a recombinant virus that expresses luciferase (VV-Luc) [54] and organs were extracted at 1dpi. Brains and spleens were fixed O/N with 4% of PFA, cryoprotected with 20% sucrose in PBS and frozen for cryosectioning. Brains and spleens of some animals were taken to titrate viral yields at 5 dpi. In these cases, brains were homogenated in 1 mL of PBS, centrifuged 10 min at 8,000×g and viral titres in the supernatants were quantified by plaque assay in BHK21 cells.

### Immunofluorescence of brain sections

Brains were cryosectioned at ∼15 µm and the resulting slides were post-fixed with 4% PFA for 20′ at RT, treated with 50 mM of NH_4_Cl, permeabilized with 0.2% Triton X-100, blocked in 5% of BSA and incubated O/N at 4°C with the indicated antibodies in a wet chamber. Primary antibodies used were: anti-phospho eIF2α (Cell Signalling, 1∶250 dilution), anti-SINV capsid (1∶500) and anti-VVp14 (1∶500). Slides were then incubated with secondary antibodies coupled to Alexa 488 or 595 for 2 h at RT, washed with PBS-0.1% Triton X-100, stained with DAPI and mounted. Slides were examined and photographed in a Leika confocal microscope.

### Metabolic labeling and Immunoblot analysis

Cells growing in 24 well plates were infected with the indicated virus at a moi of 25 pfu/cell. At the indicated times, cultures were labeled with 25 µCi/ml of Met+Cys (NEB) for 30′, washed with DMEN lacking serum and lysed in sample buffer as described previously. Samples were analyzed in a 12% SDS-PAGE, soaked in 1 M sodium salicylate, dried and exposed to X-rays films (AGFA). For immunoblot, proteins were transferred to nitrocellulose membrane and probed with the indicated antibodies: anti-PKR (1∶250, Santa Cruz Biotech.), anti-phosphoeIF2α (1∶500, Invitrogen), anti-eIF2 (1∶500 Santa Cruz) and anti-β-actin (1∶2000, Sigma). For PKR detection, protein transfer was carried out O/N at 4°C in a wet transfer apparatus (BIORAD), whereas a semi-dry transfer (BIORAD) was used for detection of eIF2 and b-actin proteins. To detect phosphoeIF2α, blots were first probed with anti-phosphoeIF2α antibody, incubated with mild stripping buffer (0.1 M NaOH, 15′) and then probed with anti-total eIF2α antibody. Blots were revealed by ECL as described previously [29].
